# Supplementary material for: The Global Prevalence and Associated Factors of Burnout among Emergency Department Healthcare Workers and the Impact of the COVID-19 Pandemic: A Systematic Review and Meta-Analysis
Source: Healthcare (Basel). 2023 Aug 7;11(15):2220. doi: 10.3390/healthcare11152220 (PMC10418606; doi:10.3390/healthcare11152220)
Supplement: Supplementary file 1 [file healthcare-11-02220-s001.zip › Supplementary Table 1.pdf]

Supplementary Table 1. The detailed search strategy employed in each of the searched databases

No.	Search	Results
**PubMed**		
**#1**	"emegency medicine" OR "emergency care" OR "emergency healthcare" OR "emergency nurses" OR "emergency physicians" OR "emergency medical technicians" OR "Emergency Medicine"[Mesh]) OR "Emergency Medical Services"[Mesh]	32,012
**#2**	burnout OR depersonalization OR "personal accomplishment" OR "emotional exhaustion" OR "Burnout, Psychological"[Mesh]	28,663
**#3**	#1 AND #2	404
**Scopus**		
**#1**	TITLE-ABS-KEY("emegency medicine") OR TITLE-ABS-KEY("emergency care") OR TITLE-ABS-KEY("emergency healthcare") OR TITLE-ABS-KEY("emergency nurses") OR TITLE-ABS-KEY("emergency physicians") OR TITLE-ABS-KEY("emergency medical technicians")	59,102
**#2**	TITLE-ABS-KEY(burnout) OR TITLE-ABS-KEY(depersonalization) OR TITLE-ABS-KEY("personal accomplishment") OR TITLE-ABS-KEY("emotional exhaustion")	73,093
**#3**	#1 AND #2	831
**Web of Science**		
**#1**	ALL=("emegency medicine") OR ALL=("emergency care") OR ALL=("emergency healthcare") OR ALL=("emergency nurses") OR ALL=("emergency physicians") OR ALL=("emergency medical technicians")	41,005
**#2**	ALL=(burnout) OR ALL=(depersonalization) OR ALL=("personal accomplishment") OR ALL=("emotional exhaustion")	31,064
**#3**	#1 AND #2	141
**EMBASE**		
**#1**	"emegency medicine":ab,ti,kw OR "emergency care":ab,ti,kw OR "emergency healthcare":ab,ti,kw OR "emergency nurses":ab,ti,kw OR "emergency physicians":ab,ti,kw OR "emergency medical technicians":ab,ti,kw	33,049
**#2**	emergency medicine'/exp	59,983
**#3**	#1 OR #2	55,395
**#4**	burnout:ab,ti,kw OR depersonalization:ab,ti,kw OR "personal accomplishment":ab,ti,kw OR "emotional exhaustion":ab,ti,kw	29,553
**#5**	burnout'/exp	34,444
**#6**	#4 OR #5	37,885
**#7**	#3 AND #6	279
**PsycINFO**		
**#1**	"emegency medicine" OR "emergency care" OR "emergency healthcare" OR "emergency nurses" OR "emergency physicians" OR "emergency medical technicians"	56,731
**#2**	burnout OR depersonalization OR "personal accomplishment" OR "emotional exhaustion"	91,932

**#3**	#1 AND #2	119
**EBSCOhost - Academic Search Complete**		
**#1**	AB "emegency medicine" OR AB "emergency care" OR AB "emergency healthcare" OR AB "emergency nurses" OR AB "emergency physicians" OR AB "emergency medical technicians"	12,483
**#2**	AB burnout OR AB depersonalization OR AB "personal accomplishment" OR AB "emotional exhaustion"	17,739
**#3**	#1 AND #2	77
**Google Scholar**		
**With all of the words**	emergency burnout	
**With the exact phrase**		
**With at least one of the words**	burnout depersonalization "personal accomplishment" "emotional exhaustion"	
**Total**	Only the first 200 records were selected	200
