# Supplementary figures and images for: The Global Prevalence and Associated Factors of Burnout among Emergency Department Healthcare Workers and the Impact of the COVID-19 Pandemic: A Systematic Review and Meta-Analysis
Source: Healthcare (Basel). 2023 Aug 7;11(15):2220. doi: 10.3390/healthcare11152220 (PMC10418606; doi:10.3390/healthcare11152220)

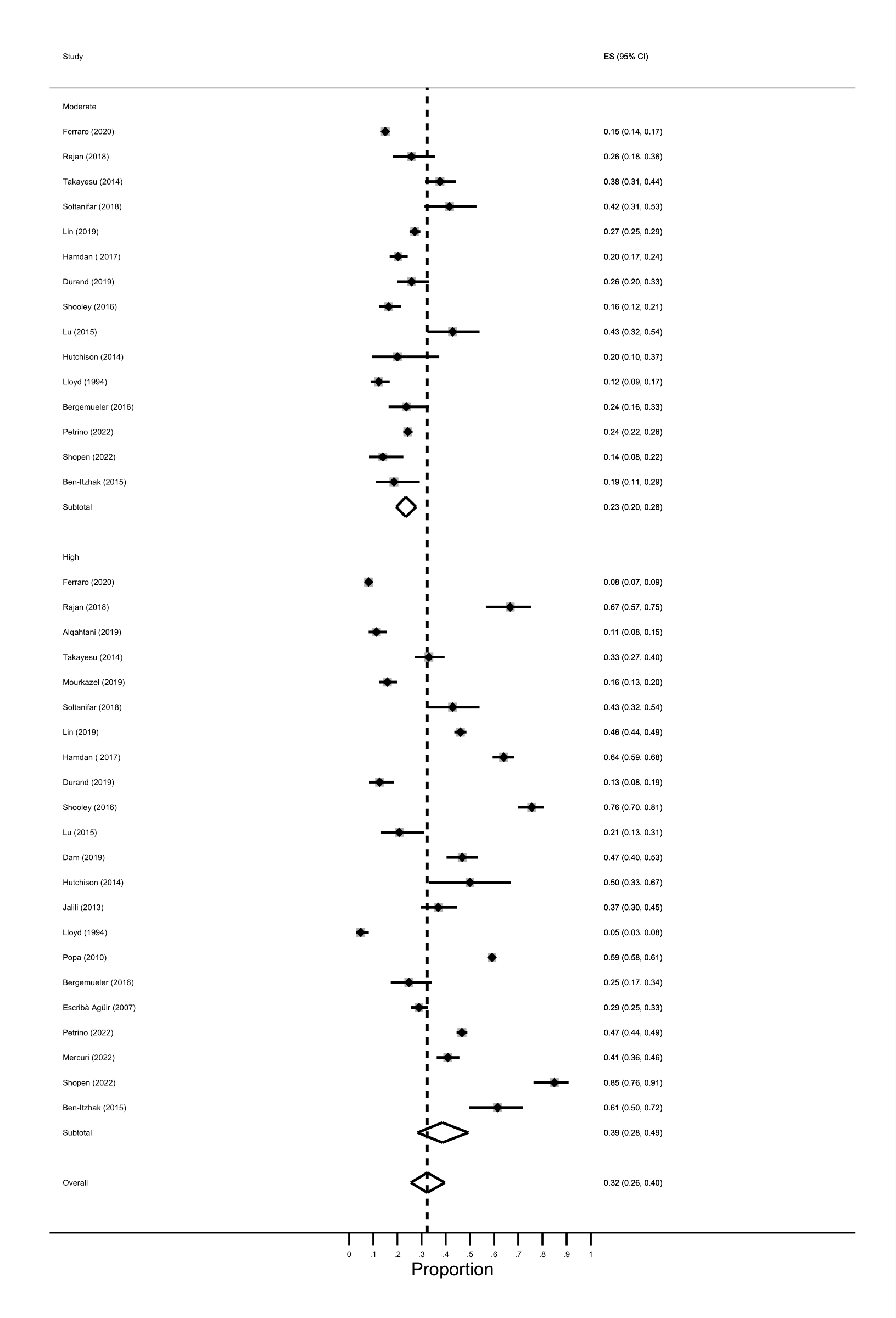

Supplement: Supplementary file 1 [file healthcare-11-02220-s001.zip › Supp Fig 1.tiff]

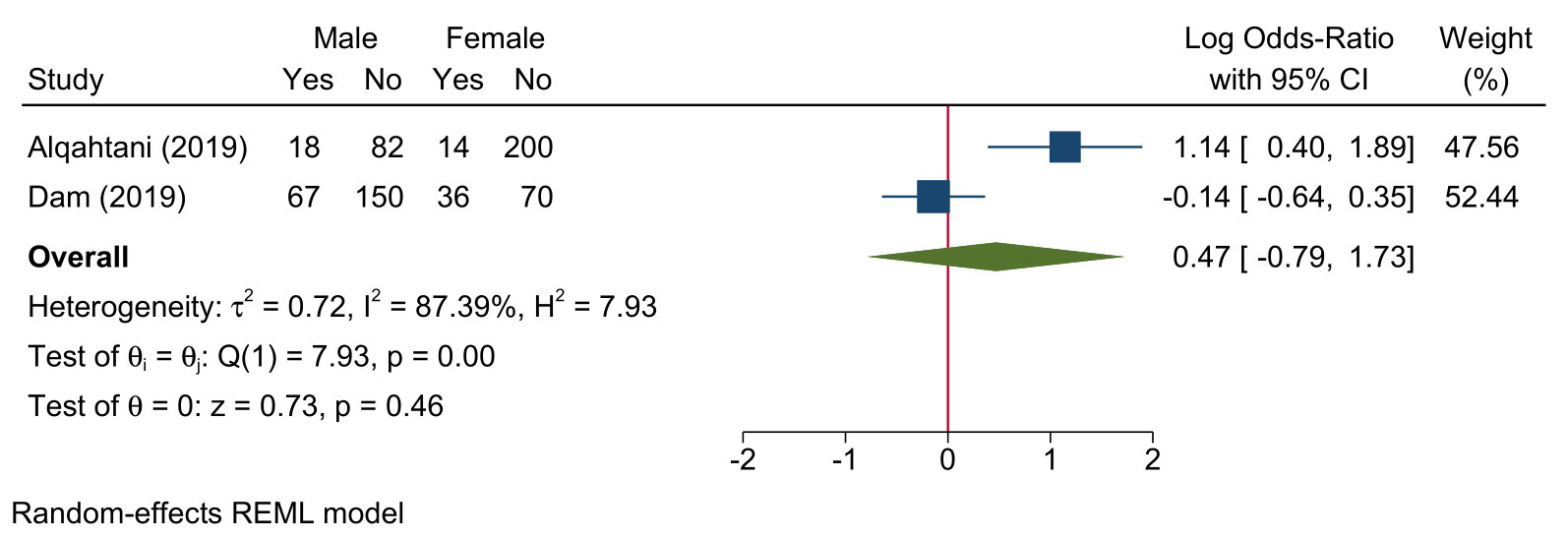

Supplement: Supplementary file 1 [file healthcare-11-02220-s001.zip › Supp Fig 10.tiff]

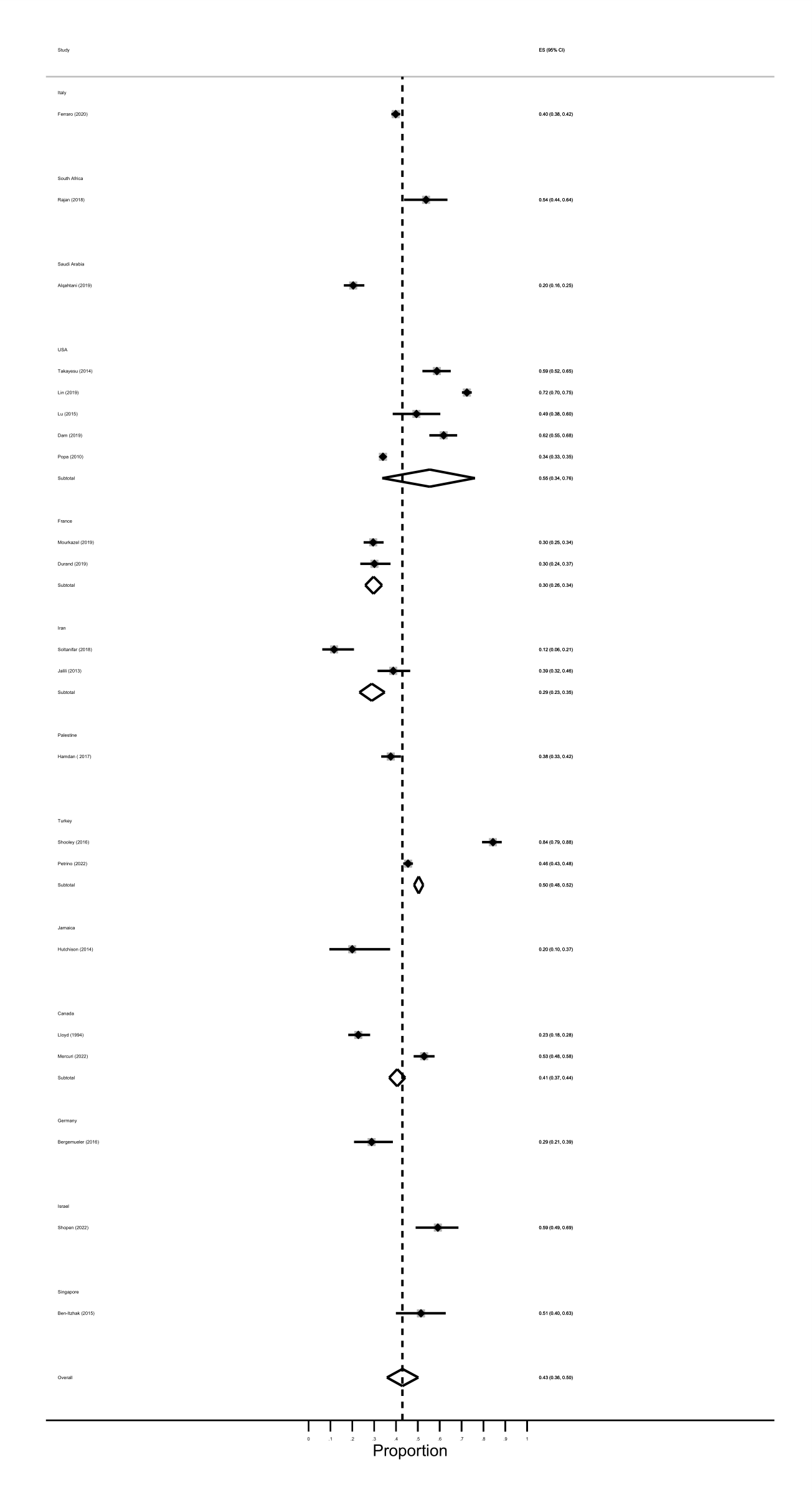

Supplement: Supplementary file 1 [file healthcare-11-02220-s001.zip › Supp Fig 11.tiff]

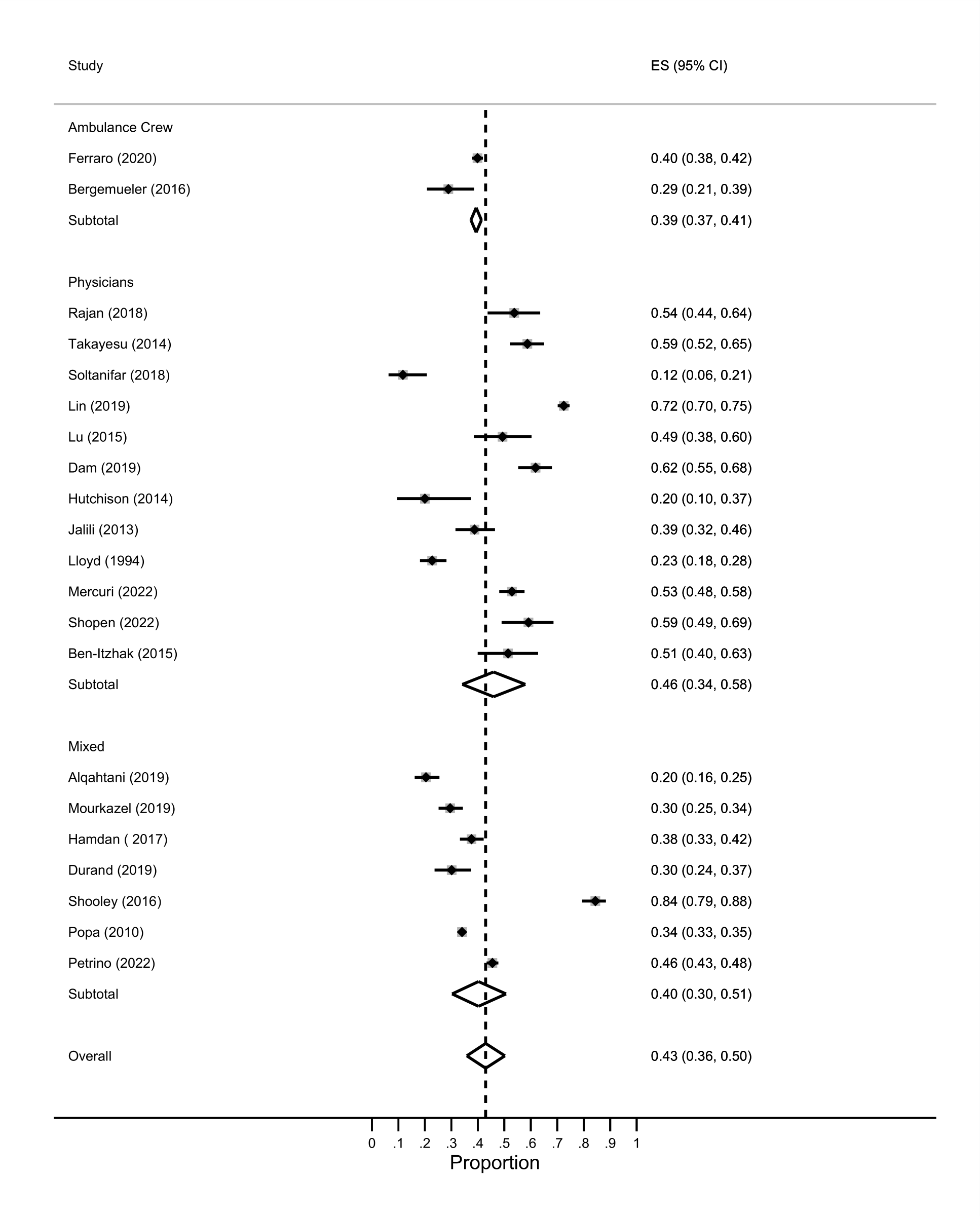

Supplement: Supplementary file 1 [file healthcare-11-02220-s001.zip › Supp Fig 12.tiff]

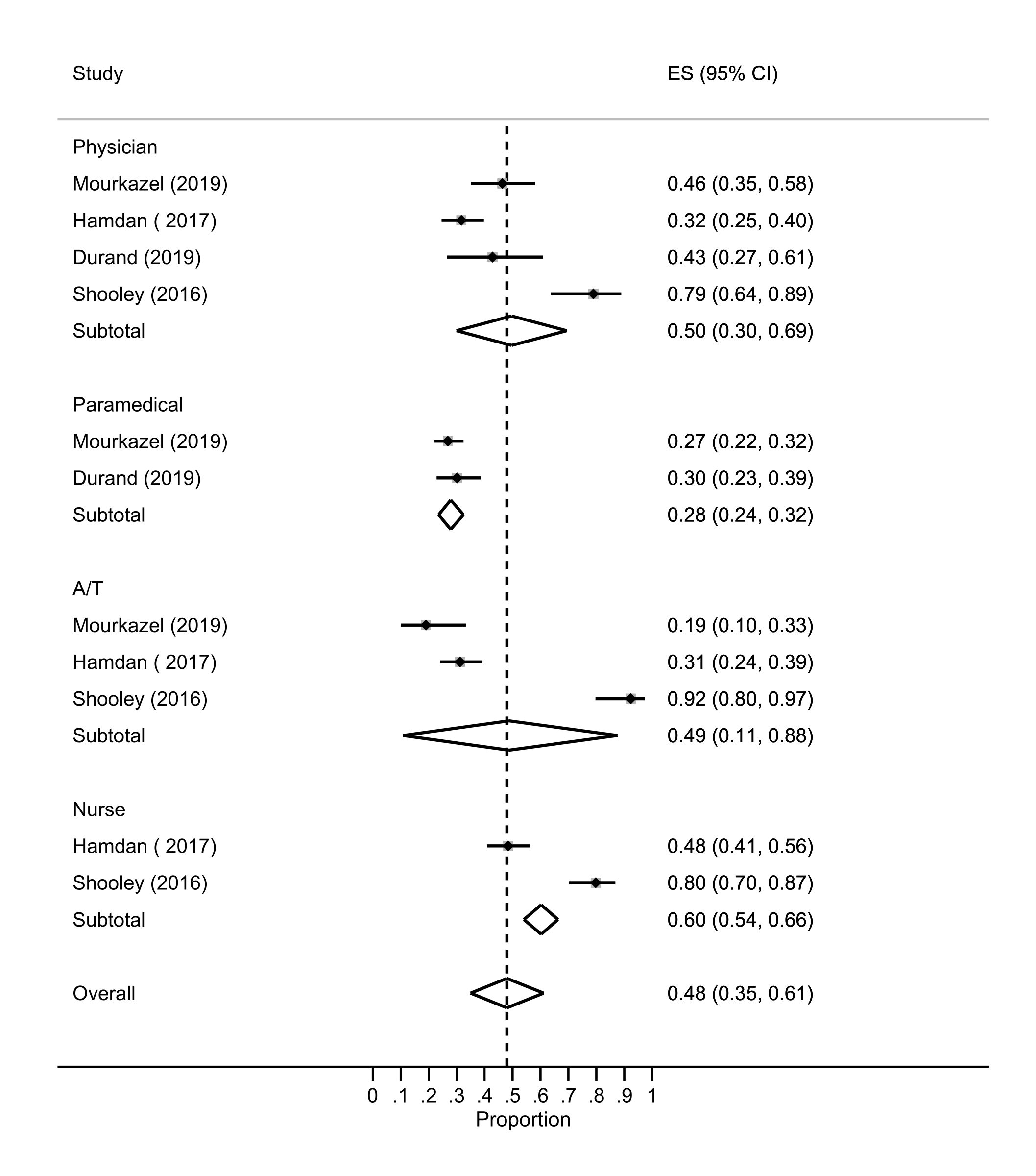

Supplement: Supplementary file 1 [file healthcare-11-02220-s001.zip › Supp Fig 13.tiff]

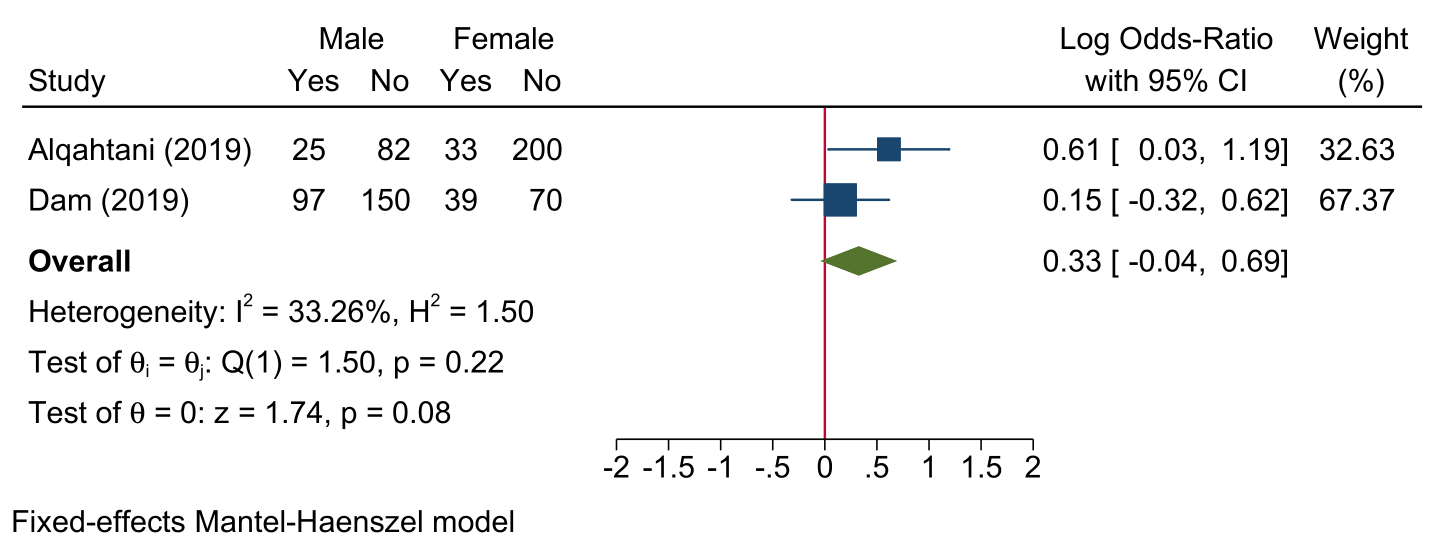

Supplement: Supplementary file 1 [file healthcare-11-02220-s001.zip › Supp Fig 15.tiff]

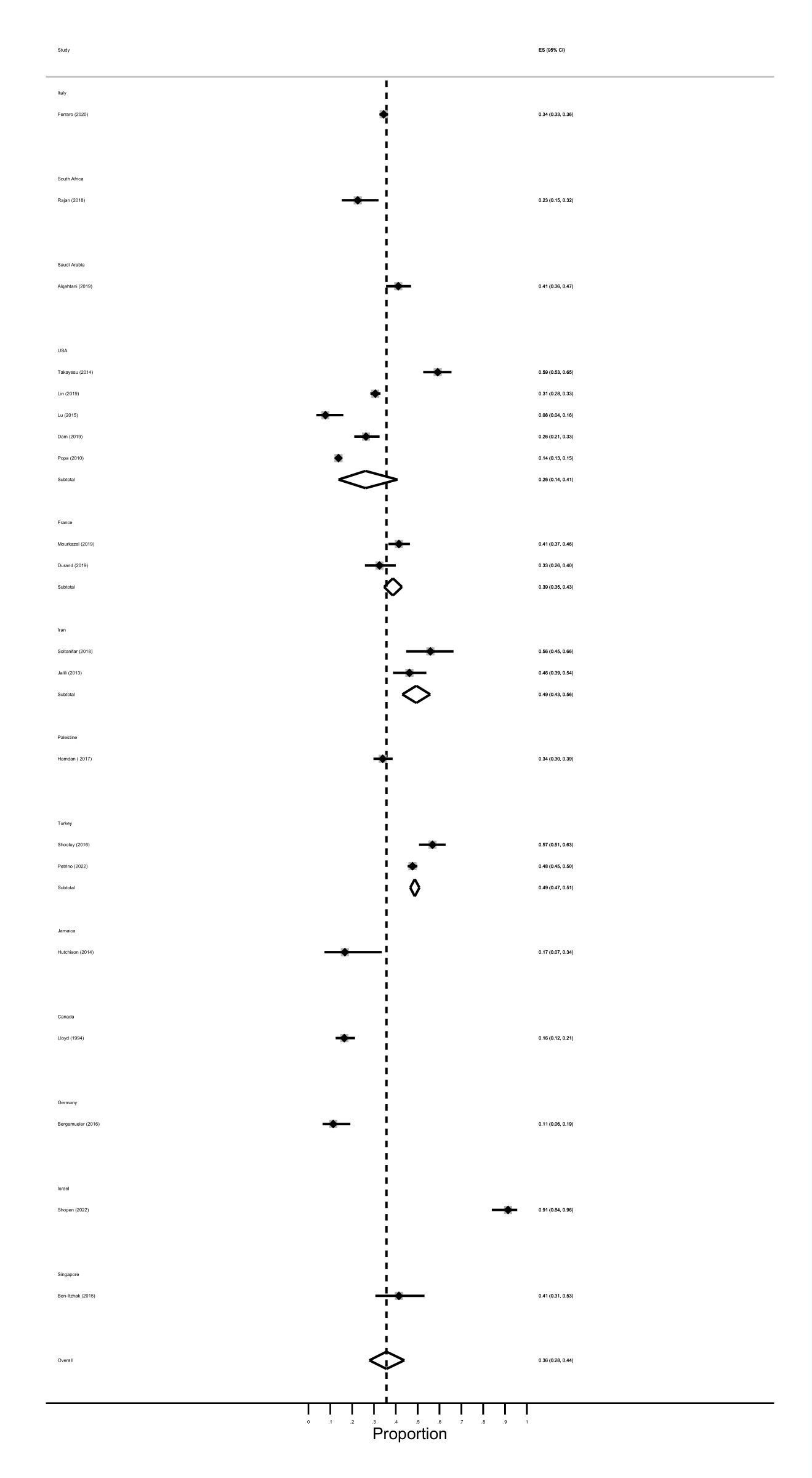

Supplement: Supplementary file 1 [file healthcare-11-02220-s001.zip › Supp Fig 16.tiff]

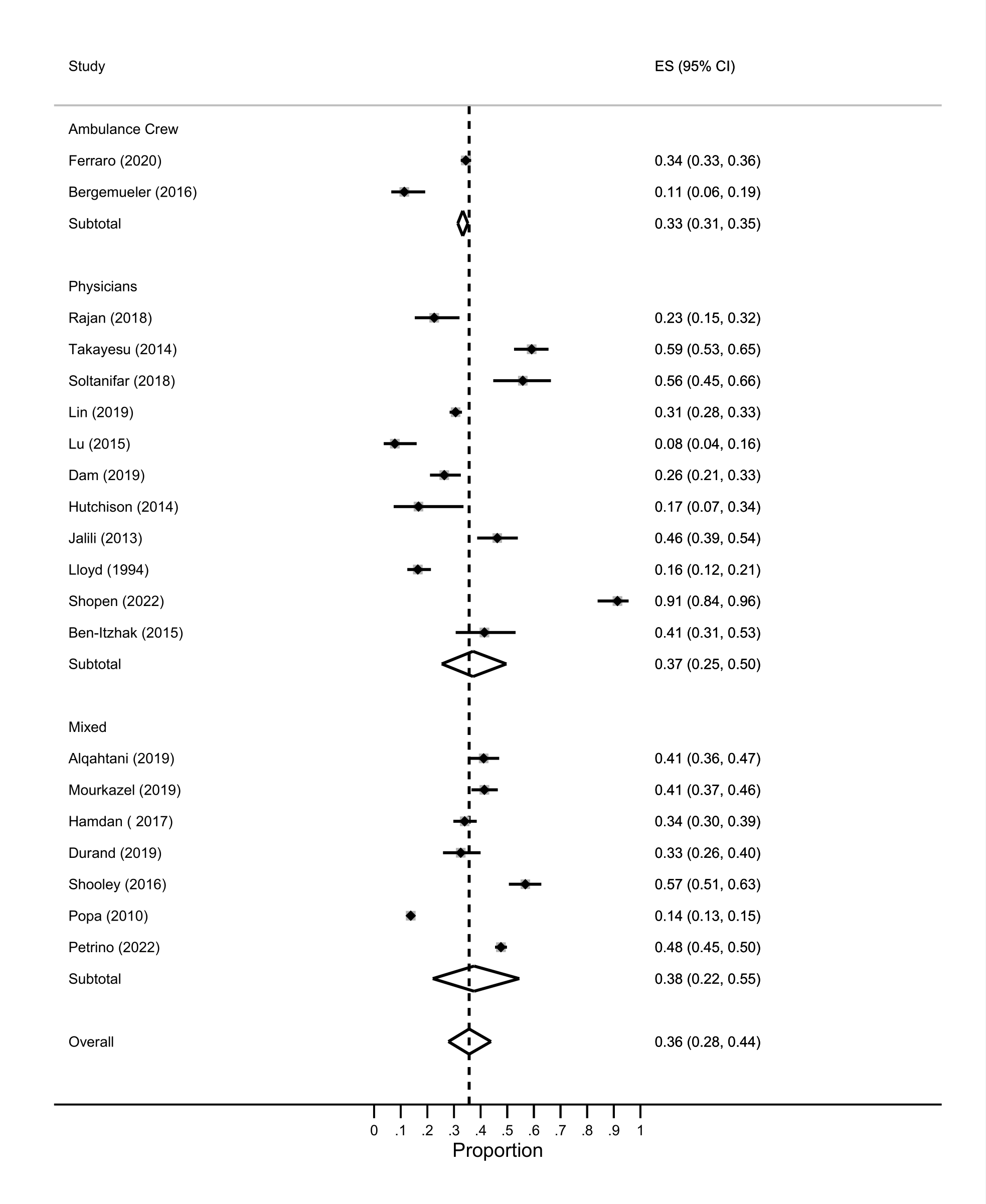

Supplement: Supplementary file 1 [file healthcare-11-02220-s001.zip › Supp Fig 17.tiff]

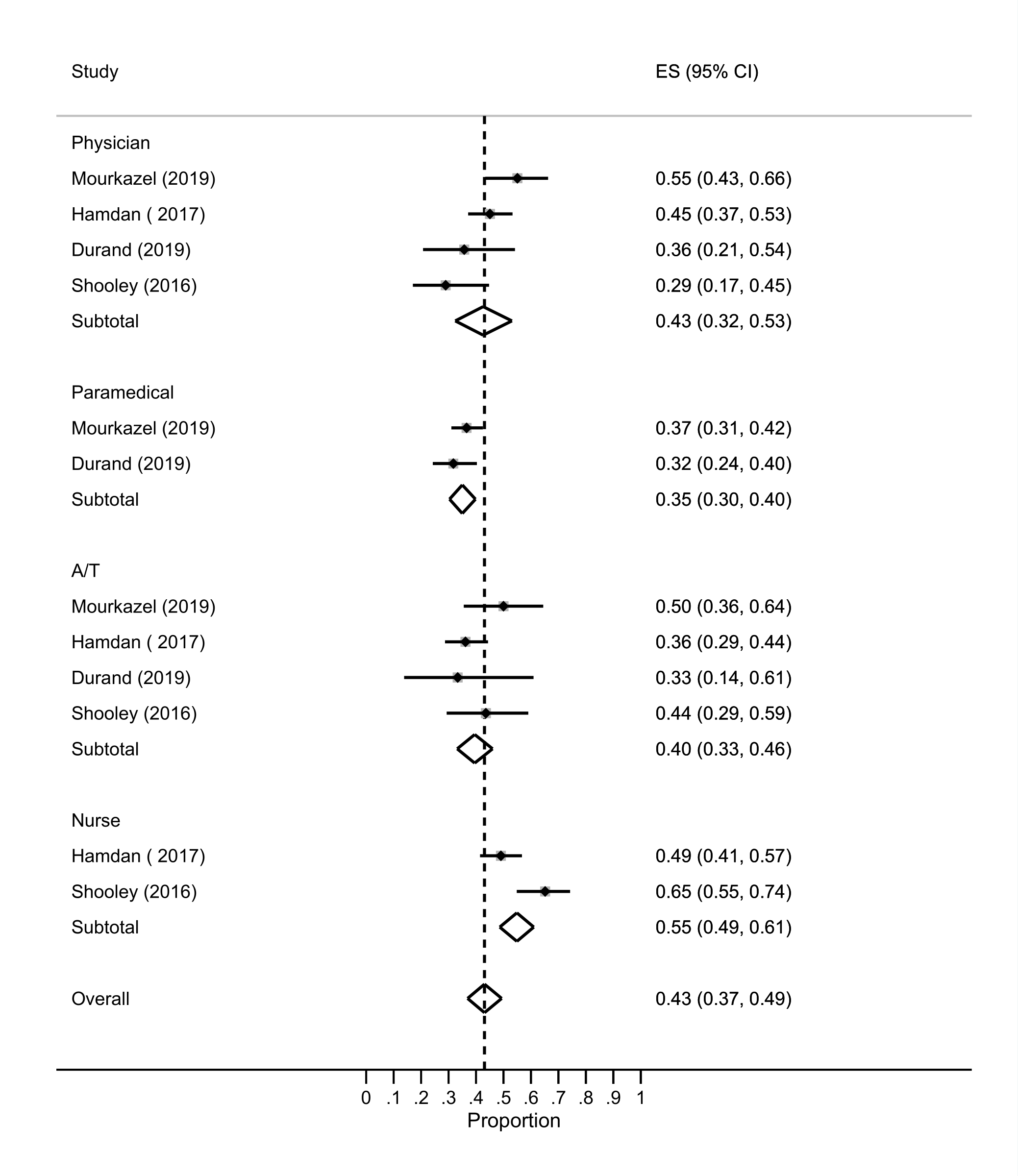

Supplement: Supplementary file 1 [file healthcare-11-02220-s001.zip › Supp Fig 18.tiff]

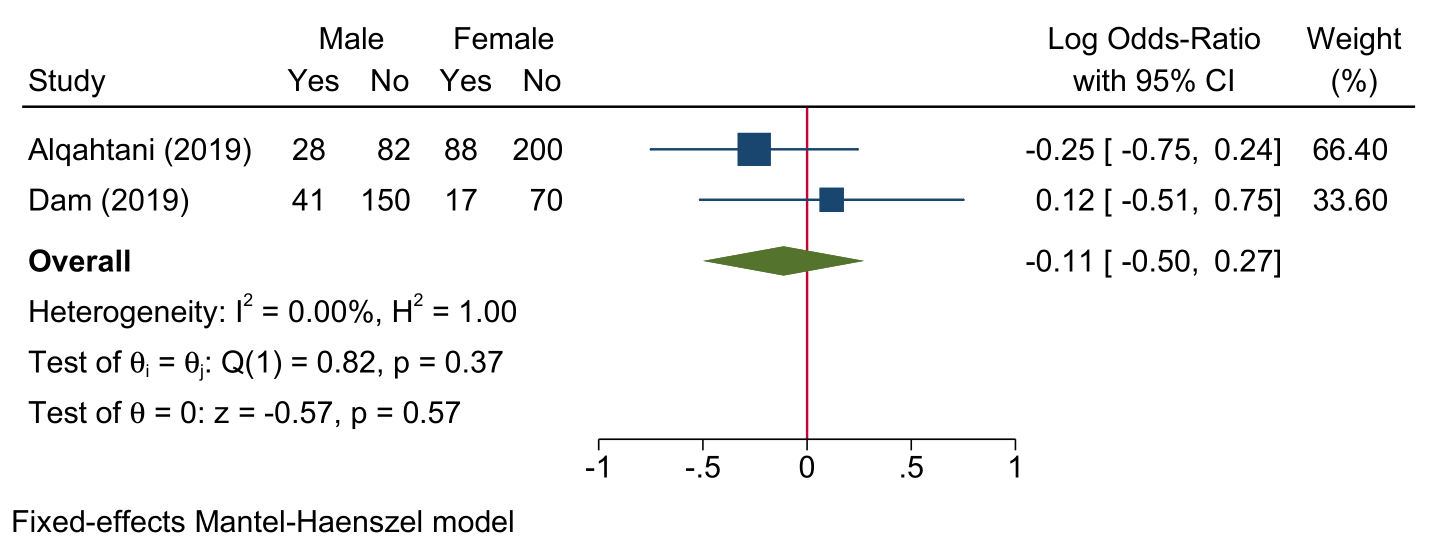

Supplement: Supplementary file 1 [file healthcare-11-02220-s001.zip › Supp Fig 19.tiff]

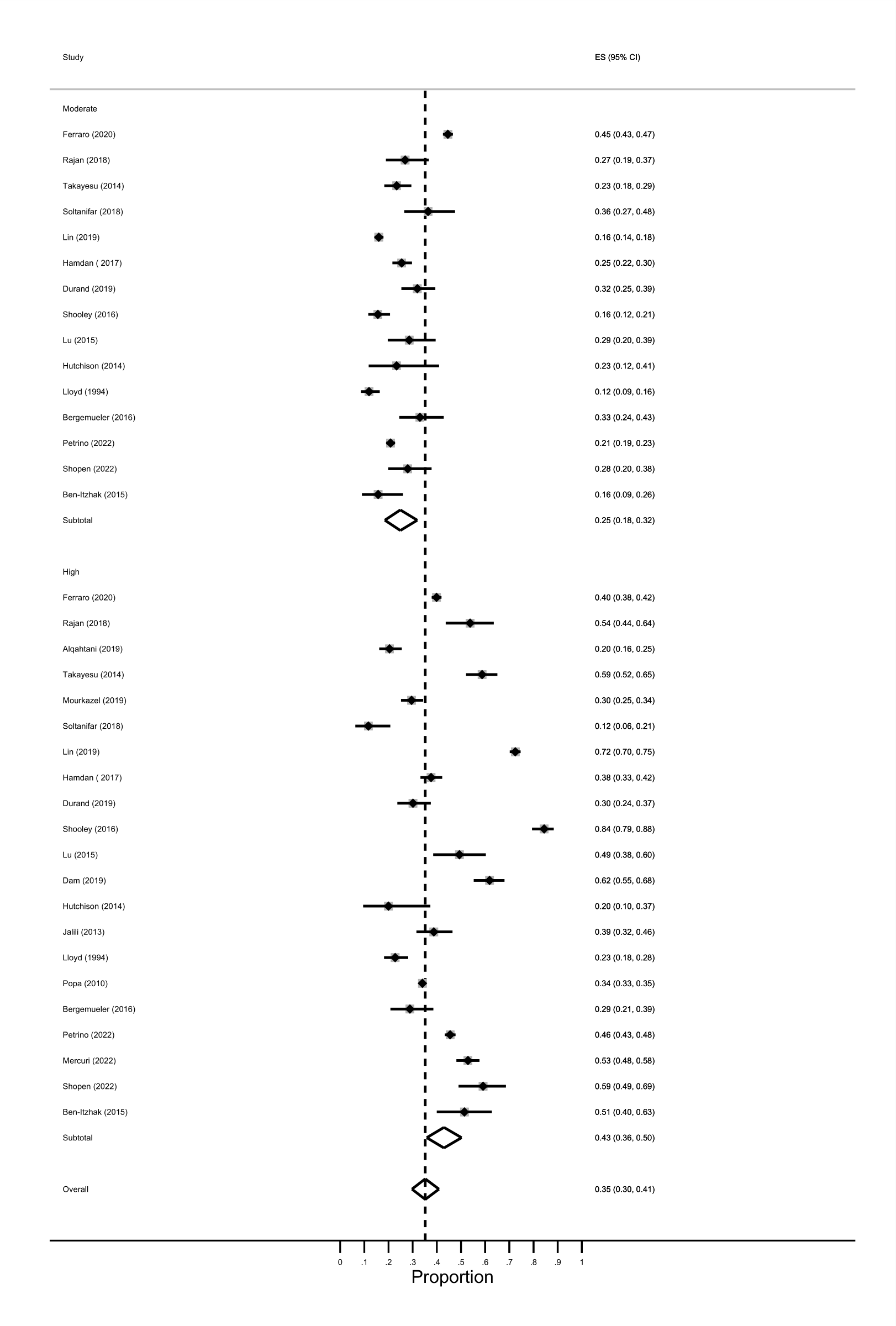

Supplement: Supplementary file 1 [file healthcare-11-02220-s001.zip › Supp Fig 2.tiff]

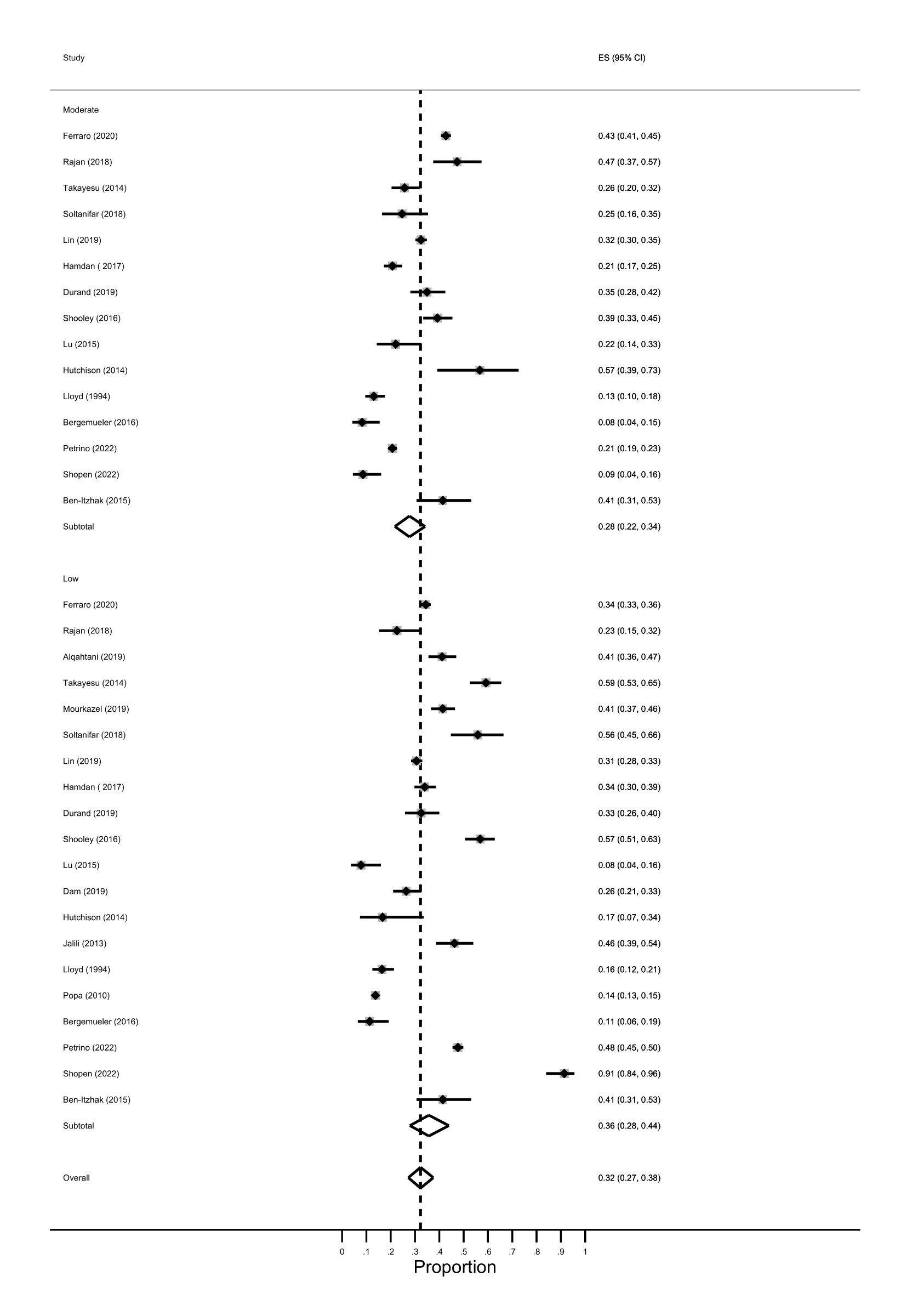

Supplement: Supplementary file 1 [file healthcare-11-02220-s001.zip › Supp Fig 3.tiff]

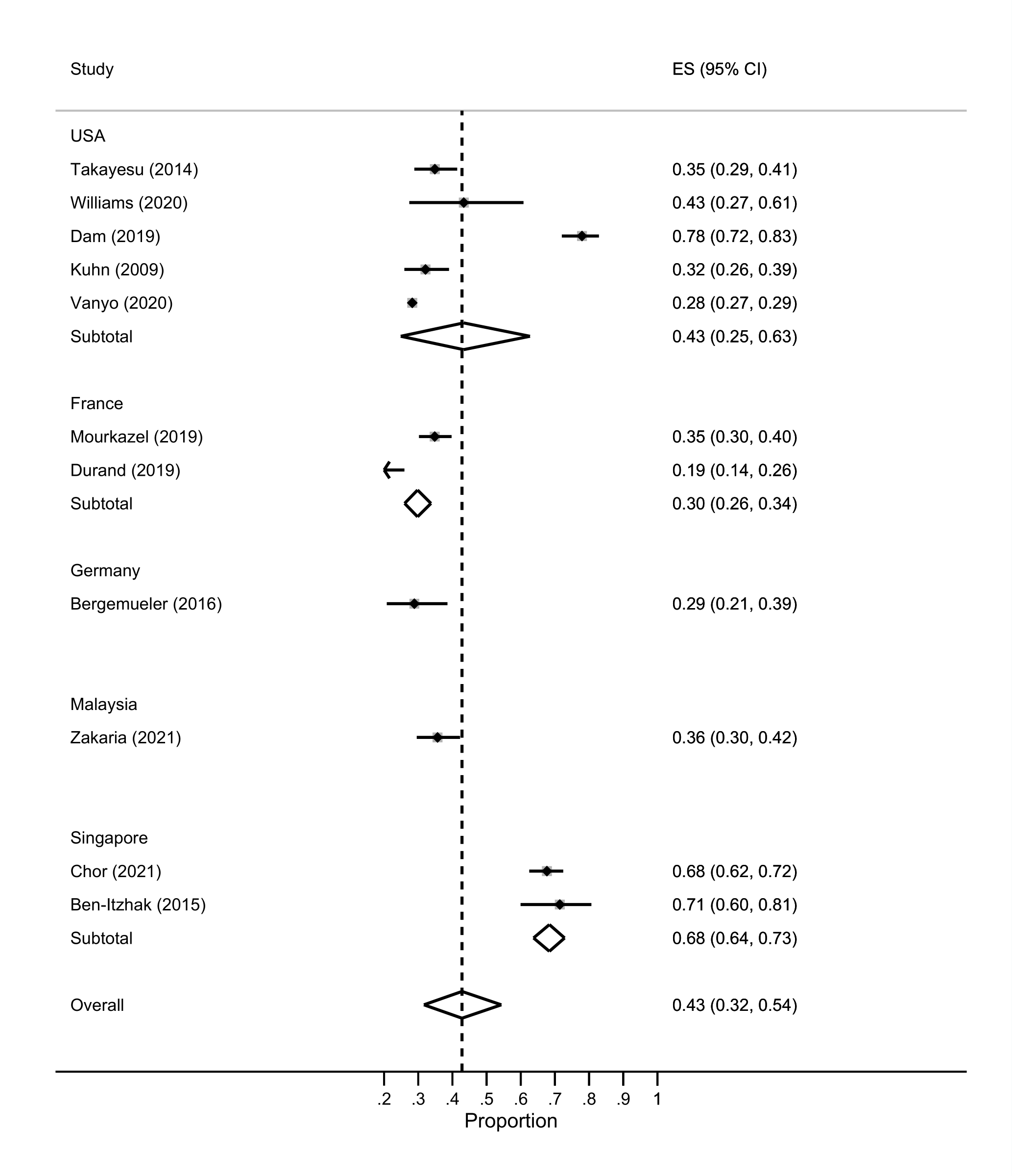

Supplement: Supplementary file 1 [file healthcare-11-02220-s001.zip › Supp Fig 4.tiff]

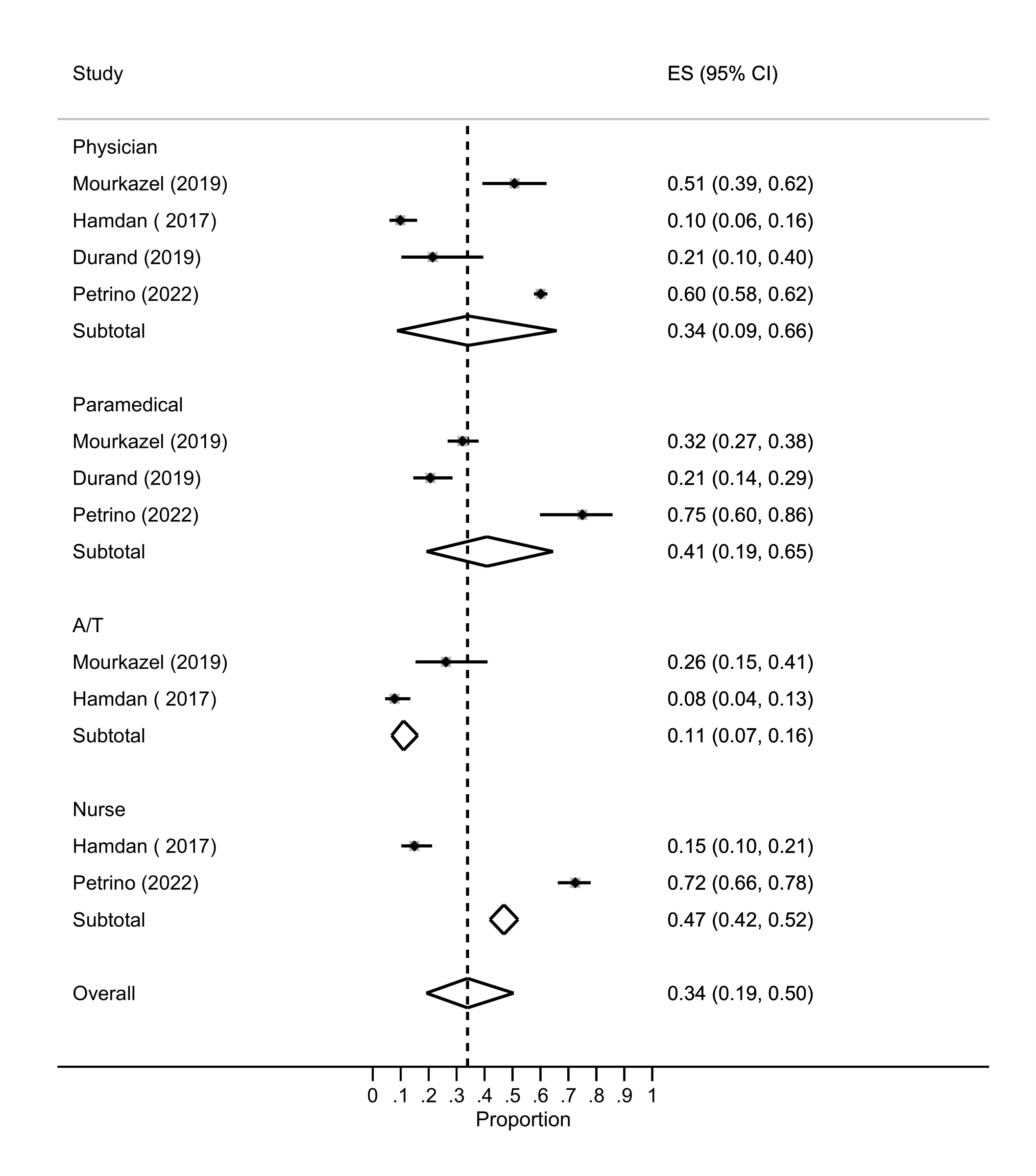

Supplement: Supplementary file 1 [file healthcare-11-02220-s001.zip › Supp Fig 5.tiff]

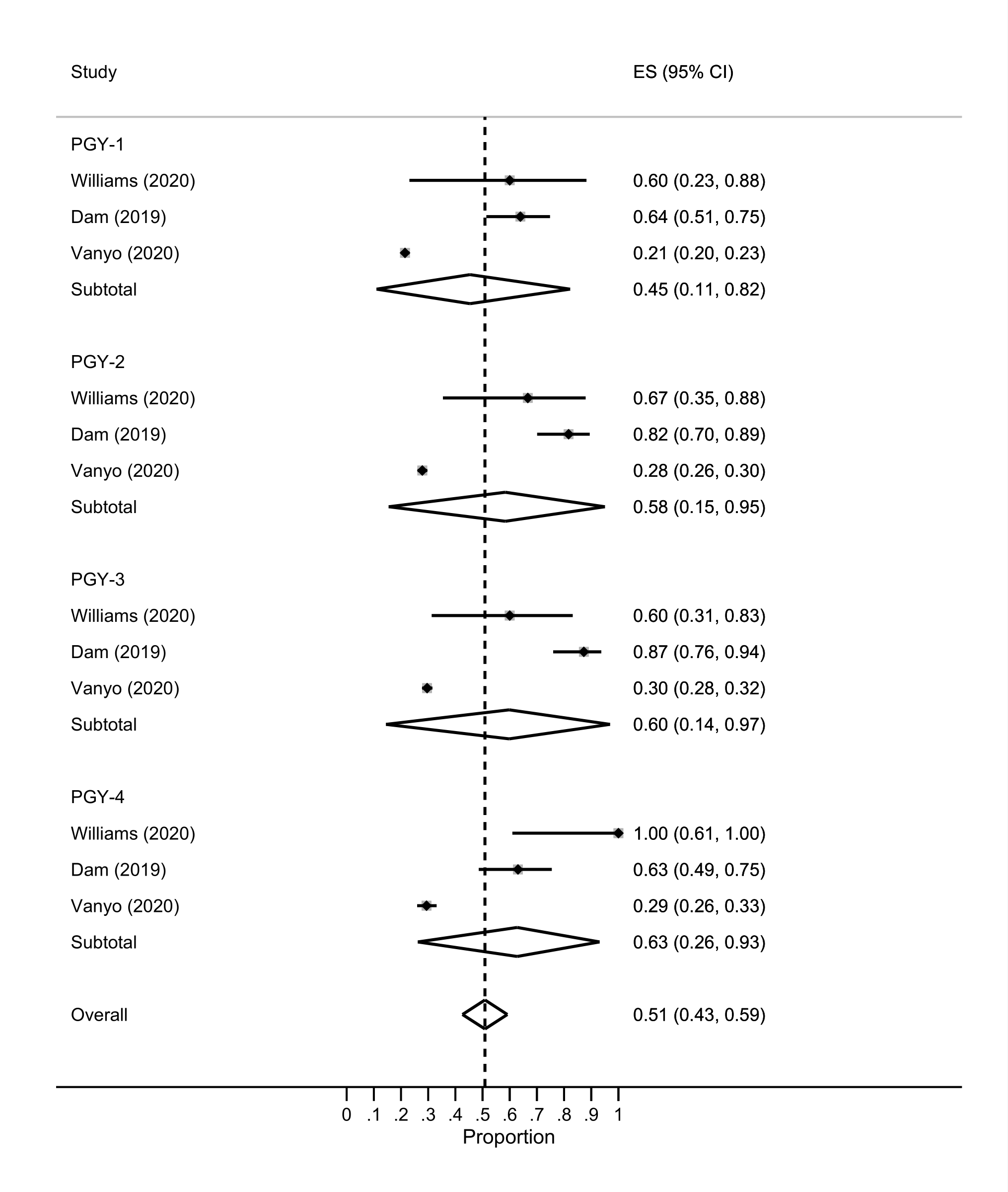

Supplement: Supplementary file 1 [file healthcare-11-02220-s001.zip › Supp Fig 6.tiff]
